# Spatial evolution in temporal dynamics of hemodynamic response function in human superior colliculi with ultra-high-resolution MRI at 9.4T

**DOI:** 10.3389/fnins.2026.1741923

**Published:** 2026-04-16

**Authors:** Nooshin J. Fesharaki, Artemy Vinogradov, David Ress, Jung Hwan Kim

**Affiliations:** 1 Department of Neurosurgery, University of Texas Health Science Center at Houston, Houston, TX, United States; 2 Department of Neuroscience, Baylor College of Medicine, Houston, TX, United States

**Keywords:** blood oxygenation level dependent, BOLD positive HRFs, functional magnetic resonance imaging, hemodynamic response function, negative HRF, neurovascular dynamics, spatial gradient, superior colliculi

## Abstract

The superior colliculus (SC) plays a crucial role in multisensory integration, visual information processing, saccadic target selection, visual selective attention, and decision making. In particular, the SC has a key role in oculomotor coordination, following a rostro-caudal organization. The rostral SC, which corresponds to foveal representation, is linked to fixation, microsaccades, smooth pursuit, and vergence adjustments. In contrast, the caudal SC, representing more peripheral visual field, is associated with the large gaze shifts (saccades). However, evidence regarding whether this functional gradient is preserved in the human SC remains limited. In this study, we employed a sequence-following visual-motor task to specifically engage SC activity. We measured blood oxygenation level dependent (BOLD) functional magnetic resonance imaging (fMRI) responses to brief neural activity, known as hemodynamic response function (HRF). We showed a spatial gradient of the BOLD positive HRFs (pHRF) along the rostro-caudal axis of the SC. The pHRF was primarily located in the rostral SC, and it gradually weakened toward the caudal SC, where negative HRF (nHRF) was often observed. The systematic rostro-caudal evolution of HRFs were consistent both within and across subjects, consistent with results from previous electrophysiological studies. Our work showed the feasibility of using ultra-high-field fMRI to non-invasively examine neurovascular dynamics in a small and deeply located subcortical structures of the human brain.

## Introduction

The midbrain–one of the phylogenetically early regions of the brain–is essential for both cognitive and sensorimotor functions. It plays a critical role in human behaviors such as the orientation of attention, arousal, and the modulation of sensory signals to the cerebral cortex. The superior colliculus (SC) is known to play a critical role in multisensory integration, visual information processing, saccade target selection, visual selective attention and decision making (Lovejoy and Krauzlis, 2010; Jun et al., 2021; Ikeda and Hikosaka, 2007; Krauzlis et al., 2013; Kim and Basso, 2008). In particular, the SC is crucial for visuomotor integration, as it transforms sensory inputs–especially visual signals from the retina and other visual areas–into motor commands that guide eye movement and orienting behavior (Stitt et al., 2013; May, 2006; Jiang et al., 2023; Durmer and Rosenquist, 2001). This layered structure supports a functional topography: the rostral SC corresponds to the foveal representation and contributes to the maintenance of fixation, the generation of microsaccades, smooth pursuit, and, to some extent, vergence adjustments (Hafed et al., 2009; Bergeron and Guitton, 2000). Neural activity in the rostral SC enables the fine control of gaze required to maintain accurate fixation and to generate subtle corrections when the eyes deviate from a visual target. In contrast, the caudal SC is involved in larger gaze shifts, initiating and guiding saccades to more peripheral locations in the visual field (Zubricky and Das, 2025; Sugiuchi et al., 2005). Because of these central roles, dysfunction in the SC–whether resulting from traumatic brain injury or neurodegenerative diseases–has been associated with impairments in visual attention, abnormal eye-movement control, and disrupted sensory integration (Burnett et al., 2004; Moro et al., 2020). Therefore, understanding the functional organization and the neurovascular dynamics of the human SC is fundamental to elucidating its critical role in human visuomotor control and relative clinical conditions.

Functional magnetic resonance imaging (fMRI) has become a powerful and widely used non-invasive tool for detecting human brain activity. Blood-oxygen-level-dependent (BOLD) fMRI experiments, in particular, offer promise for addressing the current gap in our understanding of the human brain's neurovascular response non-invasively (Huneau et al., 2015; Ogawa et al., 1992). BOLD fMRI indirectly measures hemodynamic changes associated with localized neural activity. Particularly, the BOLD hemodynamic response function (HRF) describes the temporal evolution of the BOLD signal following brief neural activity (typically lasting a few seconds). The BOLD HRF is a valuable non-invasive approach to assess neurovascular coupling in human brain, as it reflects dynamics of local changes in oxygen uptake and blood flow (Boynton et al., 1996; Logothetis et al., 2001). In mammals, the positive HRF (pHRF) typically exhibits three phases: (1) a hyperoxic peak at 4–8 s after the onset, (2) an undershoot following the peak, and 3) a damped oscillatory ringing back to the baseline.

Although fMRI techniques offer potential for advancing our understanding of neurovascular function in the SC, studying this structure remains challenging because of its deep anatomical location, small size, and close proximity to large blood vessels–all of which contribute to substantial physiological noise in fMRI data (Schumann et al., 2018; Limbrick-Oldfield et al., 2012; Barry et al., 2013; Düzel et al., 2015). Nevertheless, several studies have quantified activity in the midbrain–including the SC–using BOLD fMRI in both humans and animals (Barry et al., 2013; Lau et al., 2011; Himmelbach et al., 2007; Krebs et al., 2010; Schneider and Kastner, 2005; Wang et al., 2020; Gil et al., 2024; Shinkareva et al., 2013). In these studies, it has generally been assumed that the neurovascular response is spatially invariant across the SC.

The characterization of the BOLD pHRF in the human SC has been relatively understudied. Among the few previous studies addressing this (Field et al., 2008; Lewis et al., 2018; Wall et al., 2009), Wall et al. (2009) examined the BOLD HRF in visually responsive cortical and subcortical regions using brief visual stimuli with 3-mm isotropic voxels and 1.5-s temporal resolution (Wall et al., 2009). Despite the limited spatial resolution, they exhibited reliable pHRFs in the SC using physiological noise reduction techniques. They showed that the pHRF averaged in SC is faster and followed by an abrupt decline, distinct from those observed in both early visual cortex and the lateral geniculate nuclei (LGN). While these findings were informative, the limited spatial resolution of this study constrained the ability to fully capture hemodynamic properties of the human SC, emphasizing the need for further investigation using high-resolution imaging techniques. More recently, high spatiotemporal resolution 7T fMRI study showed significant time-to-peak differences among the SC, LGN, and visual cortex (Lewis et al., 2018). Although subcortical HRFs exhibited relatively larger variability with more noise, group average revealed consistent temporal patterns. However, because their finite-impulse-response fitting approach relied on linearity assumptions and was highly sensitive to contrast-to-noise ratio (CNR)–resulting in stronger temporal smoothing in low-CNR regions–the true temporal precision of their measurements remains uncertain.

Previously, we investigated BOLD pHRFs in the human SC, LGN, and visual cortex with high spatiotemporal resolution and compared their temporal characteristics between 9.4T and 3T (Kim et al., 2022). We measured the HRF using a model-free approach that did not rely on the linearity assumption. Our results showed that in subcortical regions, including the SC, the averaged pHRF peaks earlier and lacks the pronounced post-stimulus undershoot typically observed in cortical areas. These findings suggest that subcortical pHRFs reflect distinct vascular and neurometabolic dynamics, notably differing from their cortical counterparts. Our study demonstrated the feasibility of mapping positive BOLD responses in human subcortical nuclei, laying the groundwork for systematic investigations of neurovascular function in small and deep brain structures.

Here, we extended our analysis to investigate how BOLD HRFs evolve from the rostral to caudal portion of the SC. Previous animal studies have demonstrated that neural activity in the rostral SC supports the fine control of gaze required to maintain precise fixation and to generate subtle corrective movements following deviations from a visual target. In contrast, neurons in the caudal SC are primarily involved in encoding large-amplitude gaze shifts, reflecting their role in initiating and guiding saccades to more peripheral locations in the visual field (Sugiuchi et al., 2005; Zubricky and Das, 2025). This rostral-to-caudal organization illustrates how the SC coordinates eye movements across a wide range of amplitudes and functional demands. However, despite extensive evidence from the previous electrophysiological studies (Benavidez et al., 2021; Gandhi and Katnani, 2011), the organizational gradient of the human SC has not been extensively characterized. We acquired data using an ultra-high-field 9.4T MRI scanner with an event-related paradigm that incorporated eye movements and a sequence-following visual-motor task to engage SC activity. We characterized the HRFs with two main groups: pHRF, largely observed in the rostral portion of the SC, and negative HRF (nHRF), when present, in the caudal portion of the SC. By analyzing the spatial gradient of the BOLD HRF along the rostro-caudal axis, we found that the pHRF gradually weakened toward the caudal SC, with the averaged nHRF largely reflecting a continuation of this spatial evolution. This systematic mapping of evolution of the BOLD HRF along the rostro-caudal axis in the SC provides new insights into functional organization of human SC and advances our understanding of its role in visuomotor control.

## Materials and methods

*Participants**:* We collected data from seven adults (age: 20-60 years) with no history of neurological diseases. Our study was performed according to a protocol reviewed and approved by the Ethics Review Board of the Eberhard Karl's University, Tübingen and in agreement with the World Medical Association Declaration of Helsinki (World Medical Association, 2013). Additionally, a local physician interviewed with each subject before MR session in order to ascertain that all MR-safety-related criteria were fulfilled. Prior to the experiment, a written, informed consent form was collected for each subject. Each subject was also screened for MRI safety at the beginning of the scanning session.

**Figure 1:**
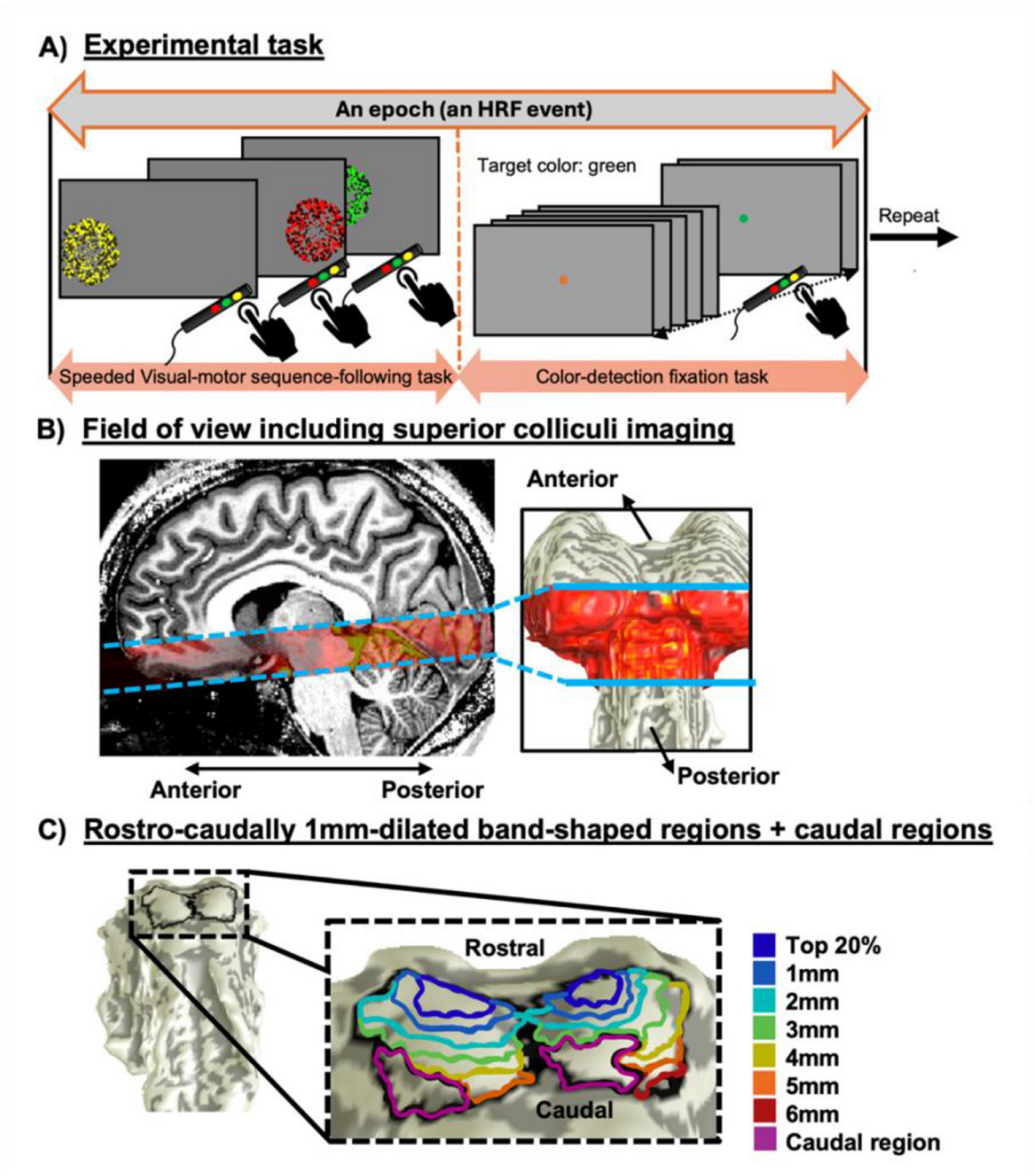
**(A)** Experimental task including epochs of a 2-s visual-motor task following by a 24.25-s color-detection fixation task. **(B)** Field of view for functional imaging from a sagittal view **(left)**, and on a subcortical surface mesh **(right)**. **(C)** An example of 1mm-thickness band-shaped regions-of-interest dilated outwards rostro-caudally from the region containing the top 20% of strong positive HRFs and the corresponding caudal regions with negative HRFs.

*Magnetic resonance imaging data collection protocols**:* Data acquisition was conducted using a 9.4T Siemens MRI scanner (Siemens Healthcare, Erlangen, Germany) integrated with the Siemens whole-body SC72 gradient system at the Max Planck Institute for Biological Cybernetics. A customized head coil with a 16-channel dual-row transmit array, paired with a 31-channel receive array was used. For each subject, we obtained a high-resolution T1-weighted anatomical reference volume using a MP2RAGE sequence: 0.6-mm isotropic voxels, TR = 6,000 ms, TE = 3 ms, TI = 800/2,000 ms, flip angle = 5°/9°. In a separate session, we then performed five T_2_*-weighted functional scans to obtain BOLD HRFs using a point spread function (PSF) corrected echo-planar imaging (EPI) sequence: 1-mm isotropic voxels, TR = 1,250 ms, TE = 21 ms, bandwidth = 1,254 Hz, duration of readout read-out train = 39.375 ms, field of view (FOV) = 210 mm, GRAPPA = 4 and 16 quasi-axial slices. The FOV was selected to encompass subcortical regions, including the SC (Figure 1B). At the beginning and end of functional scans, a set of T1-weighted structural images, referred to as an “Inplane” anatomy, was obtained with the same prescription as the functional scans using a 3D FLASH sequence with minimum TR and TE: 0.8 x 0.8 x 1 mm^3^ voxels, 24 slices and acquisition time 4 min. The Inplane were then used to align the functional data with high-resolution anatomical images.

*Stimulus**:* Subjects performed a slow event-related-design experiment during each functional scan, including 17 repetitions of two consecutive epochs of (1) a 2-s visual stimulus with concurrent motor planning and response, and (2) a slow-paced fixation task (Figure 1A). During the first epoch, three consecutive presentations of flickering colored-dot patches in one of the three colors of yellow, green, and red, were displayed on an MRI-compatible screen mounted at the back of the MRI scanner. Each colored-dot patch could be presented in one of the three circular regions with 5° radius, which were uniformly and horizontally distributed alongside the length of the screen. The presentation of patches had a random spatial order, with no sequential repetition for the same-colored patch. However, each colored-dot patch was consistently presented in the same position: yellow on the left, green in the center, and red on the right. During this 2-s time window, subjects were instructed to follow the colored-dot patches with their eye movements and quickly press three hand-held buttons in the order corresponding to the color of the presented patches on the screen. For the second epoch (24.25-s duration), subjects were asked to fixate their gaze on a fixation dot at the center of the screen with a color changing every 0.6 s and press the yellow button when the dot was green. This non-demanding, fixation-point, color-detection task was to retain a low level of attention, avoiding cognitive carryover. An additional 12-s blank time window was also included at the beginning of each functional scan to account for the scanner-related effects and hemodynamic onset transients. With five functional scans per session, we thus collected a total of 85 (17 HRFs per scan x 5 scans) HRFs per subject.

*Structural segmentation and surface modeling**:* Brainstem segmentation from the high-resolution anatomical volume was performed using a probabilistic Bayesian approach in the FreeSurfer software (Iglesias et al., 2015; Dale et al., 1999). The midbrain and portions of the thalamus were segmented through a semi-supervise approach using the ITK-SNAP application (Yushkevich et al., 2016). For surface modeling, we employed an iso-density surface-rendering technique to construct the surface between the brainstem and cerebrospinal fluid from the segmentation. The surface was further refined using a deformable-surface algorithm driven by a curvature flow (Khan et al., 2011; Bajaj et al., 2008b; Xu et al., 2006), and a single connected region for each SC was manually drawn on the refined surface. We used the same procedure to generate an interface for the cerebral aqueduct (CA).

*Depth mapping*: We computed a normalized depth coordinate between the CA interface and the posterior brainstem roof (i.e., the tectum of the rostral midbrain) surface using our watershed approach (Khan et al., 2011; Truong et al., 2020). This was performed by creating a self-reciprocal depth coordinate system in the space between the two surfaces (Bajaj et al., 2008a; Dale et al., 1999; Yushkevich et al., 2016). In this depth coordinate system, the Euclidean distance from each voxel in the volume to the two surfaces was calculated. Each voxel was also assigned a sign based on its location relative to the surface; positive if it was enclosed within the surface, and negative if it lay outside. We defined a normalized distance parameter *w*:


$$\begin{array}{l} wS_{CA}+\left(1-w\right)S_{T}=0 \end{array}$$

where the *S*_*T*_ was the signed distance from the posterior brainstem roof surface, and *S*_*CA*_ was the signed distance from the CA interface (de la Rosa et al., 2021). The normalized distance *w* was thus independent of the midbrain thickness variations with zero at the posterior brainstem roof surface and unity at the CA interface. This approach resulted in normalized depth maps consistent across subjects.

*Generating the streamlines**:* We next generated streamlines, establishing a one-to-one correspondence between vertices on the posterior brainstem roof surface and those on the CA interface. The field ∇*w* was formed by convolution with 5-point kernels in each cardinal direction, and ray tracing was performed along this field. By gradually stepping from the posterior brainstem roof surface to the CA interface in small increments, we obtained a series of trajectories (Kim and Ress, 2017; Khan et al., 2011). The tracing process was initialized along the inward surface normal at each node on the brainstem surface, and propagated in small increments, thereby yielding distances along each trajectory (Truong et al., 2020). The terminal distance values obtained at the endpoint of the trajectory were re-gridded onto the posterior brainstem roof surface to create a physical thickness map.

*Functional data preprocessing**:* For each fMRI scan, slice acquisition timing correction was performed using cubic-spline interpolation. Head-motion correction was then performed using a robust expectation-maximization method (Nestares and Heeger, 2000). Next, the functional timeseries were compensated for low-frequency temporal baseline drifts using a high-pass filter. An estimated baseline for each timeseries was obtained by smoothing it twice using a RECT-function kernel with the same duration as an HRF. The resulting baseline was then subtracted from the timeseries. Finally, the corrected data were spatially aligned on their corresponding high-resolution T1-weighted reference anatomy using the Inplane collected in each session. This fMRI analytical approach has been widely used in our previous studies to characterize BOLD signals in both cortical and subcortical brain regions (Katyal et al., 2010; Savjani et al., 2018; Katyal and Ress, 2014). Registration quality was visually inspected for each subject, with particular attention to alignment of the midbrain (Supplementary Figure S1).

*HRF data analysis**:* The timeseries data were depth-averaged along the midbrain streamlines within a physical thickness of 0.5–2 mm to minimize partial volume effects, avoiding contaminations from adjacent structures, particularly the periaqueductal gray. The averaged timeseries data were then mapped onto the posterior brainstem roof surface, such that each vertex contained 85 HRFs (17 HRFs per scan x 5 scans). The depth-averaged timeseries were upsampled to a temporal resolution of 0.1 s and baseline-corrected by subtracting a zero baseline defined as the average of its first and last time points of each HRF. Bootstrapping (*n* = 2000, random sampling with replacement) was then performed across 85 HRFs to account for variability at each time point (Efron and Tibshirani, 1994; Efron, 1987). For each vertex, we estimated the mean response and 68% confidence intervals (CI), corresponding to the standard error of the mean under the assumption of normality (Efron, 1981). Each 26.25-s HRF epoch corresponded exactly to 21 TRs, ensuring consistent temporal alignment across repetitions prior to upsampling and bootstrapping.

*HRF classification**:* Mean bootstrapped HRFs within the SC were classified into either pHRF or nHRF. The HRF was classified as a pHRF if it exhibited a significant positive peak amplitude, defined as the largest value within the first 2–14 s after the stimulus onset, with a CNR >1. The peak CNR was defined as the ratio of the mean peak amplitude to the mean of its corresponding 68% CI. However, the HRF was classified as a nHRF if it showed a significant initial negative response (INR) amplitude. The INR was defined as the magnitude of the negative BOLD response following the stimulus onset, and it was significant if its CNR, defined as the ratio of the INR to the mean of its corresponding 68% CI, was >1.

*Dilation-based regions-of-interest (ROIs)**:* To investigate the spatial dynamics of HRFs in more detail, we examined how pHRFs evolved along the rostral-caudal axis, eventually reaching the caudal region where nHRFs were observed. We created a ROI in the most rostral SC at the location of clustered strong pHRFs, specified as those with peak CNR >3 and within the top 20% of peak amplitudes. From this ROI, we generated multiple band-shaped dilation ROIs, each with a thickness of 1 mm, using a flood-fill approach (Teo et al., 1997). This was accomplished by calculating the distance from all vertices on the boundary of this ROI in the normal direction along the posterior brainstem roof surface. For example, the first band-shaped ROI was generated by spanning 0–1 mm from this ROI. The dilation procedure was then repeated to create additional concentric, 1mm-thick band-shaped ROIs extending rostro-caudally across each SC. Noteworthy, all dilated ROIs were constrained to remain entirely within the boundaries of each SC, shown in Figure 1C.

To quantify rostro-caudal trends in peak amplitude, we performed linear regression analyses separately for each SC, with normalized rostro-caudal distance as the predictor and peak amplitude of the mean pHRF within each 1-mm dilated ROI as the outcome variable. Statistical significance of the slope was assessed at *p* < 0.05, and we reported the Pearson correlation coefficients (*R*).

## Results

**Figure 2:**
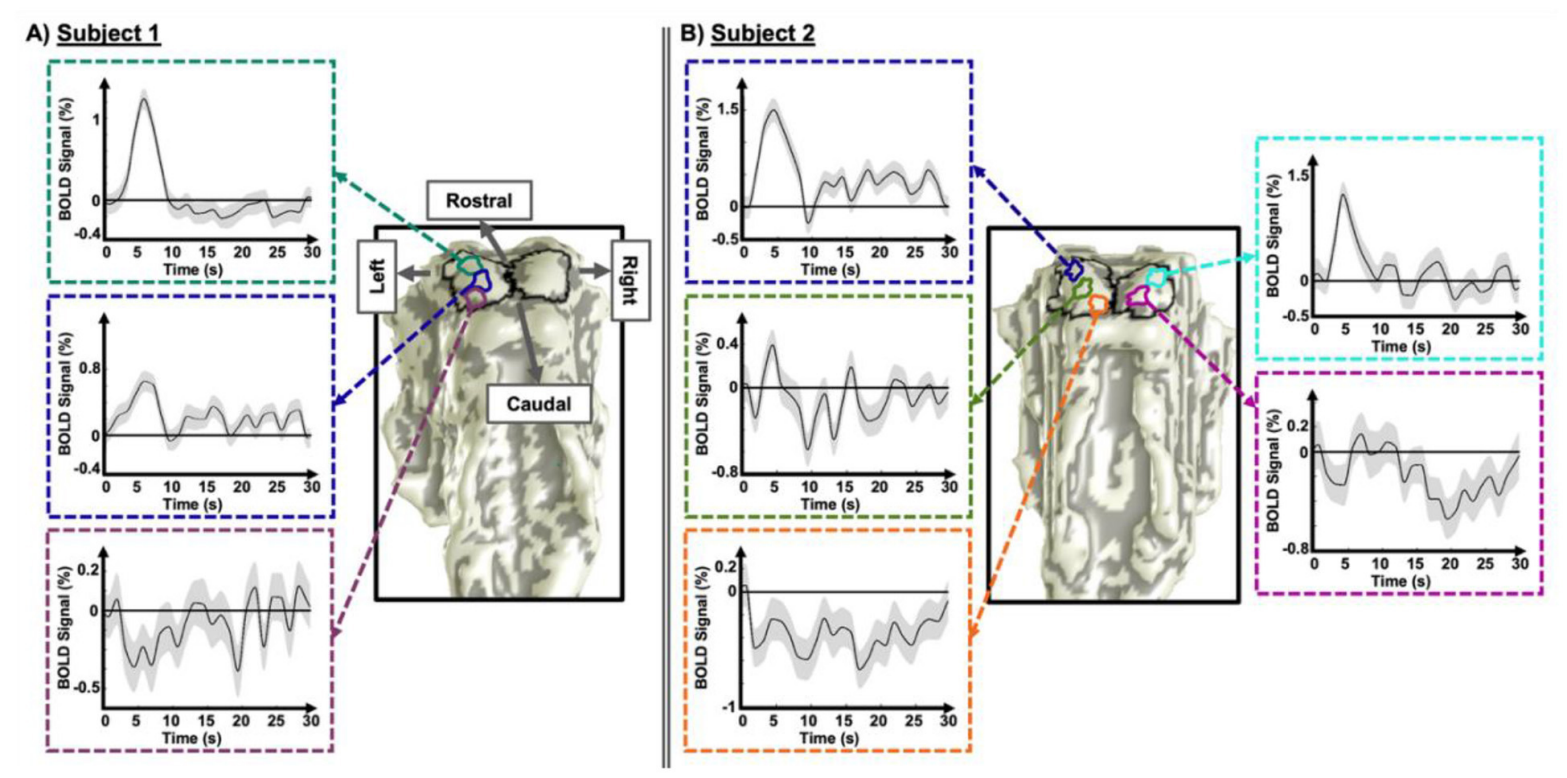
Examples of spatial variations in the dynamics of HRF in the SC for two representative subjects. **(A)** Subject 1: three ROIs are shown along the left SC from rostral (top) to caudal (bottom). **(B)** Subject 2: three ROIs are shown along the left SC from rostral (top) to caudal (bottom), along with two ROIs in the right SC (one rostral [top] and one caudal [bottom]). For each ROI, the corresponding HRF time series is displayed. Across ROIs, the positive HRF progressively weakens from rostral to caudal locations, with the emergence of negative HRF in the most caudal regions. Shaded areas represent the SEM.

*Spatial variations in HRF dynamics in the SC**:* Our examination of HRFs within the SC showed spatially organized variations in their temporal dynamics. In the most rostral SC, we observed stereotypical pHRFs resembling those widely observed in the cortex (Fesharaki et al., 2026; Kim et al., 2022; Taylor et al., 2018; Fesharaki et al., 2024), however notably faster time-to-peak (4–6 s) was observed, comparing to cortical HRFs (approximately 6–8 s). From the rostral to caudal SC, the peak amplitude of pHRFs gradually weakened (Figure 2). In the caudal SC, this change became more pronounced, often resulting in nHRFs that began with an INR followed either by a positive peak or by a return to the baseline without a discernible peak.

**Figure 3:**
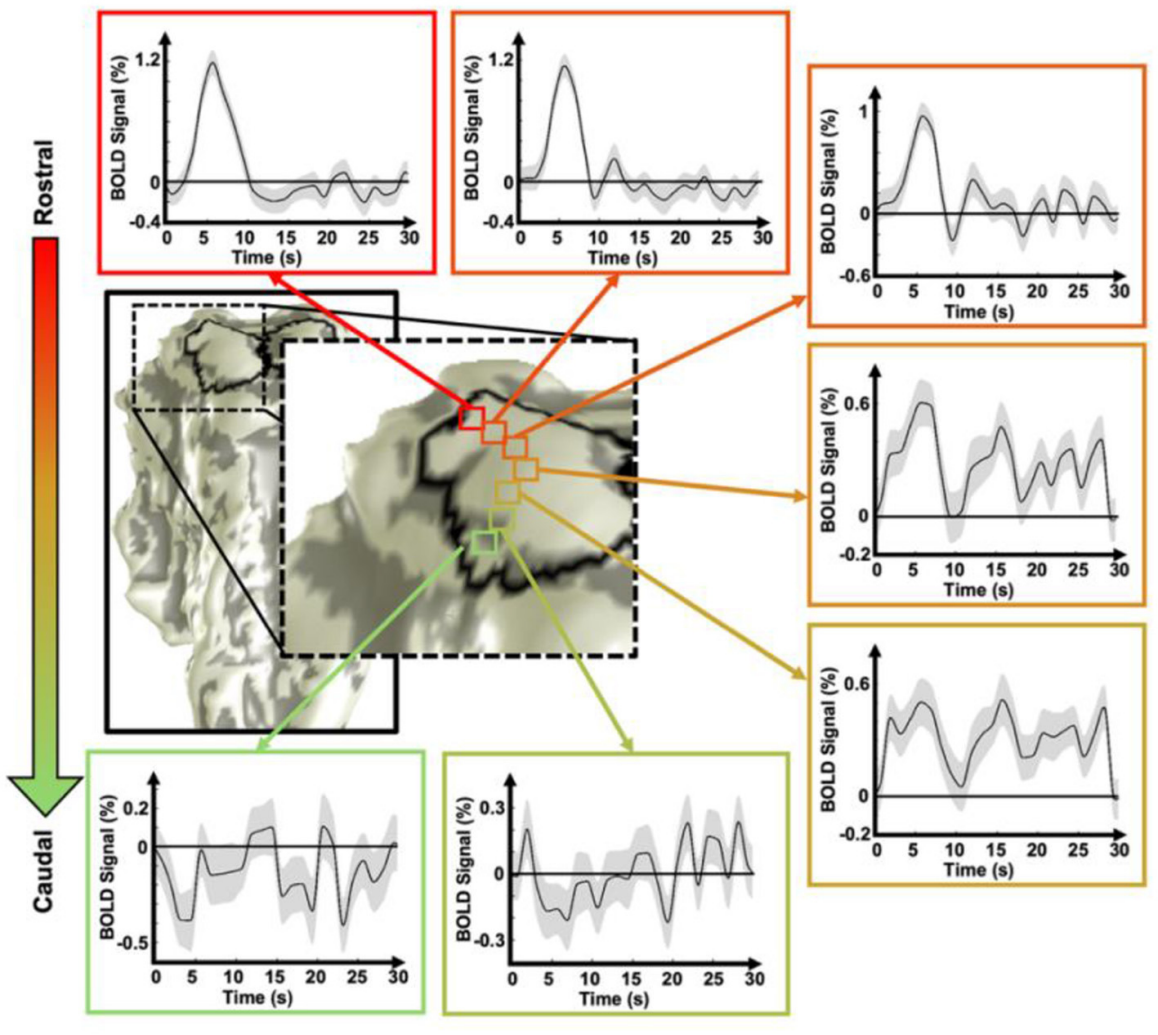
An example of voxel-wise variations in the HRF dynamics along the rostro-caudal axis in the SC. The shaded area represents the SEM.

We investigated further to show rostral-caudal gradients of HRF dynamics on a voxel-wise basis. We selected voxels aligned along the rostro-caudal axis in the SC (Figure 3). The HRF of the most rostral voxel exhibited a stereotypical pHRF, characterized by a strong peak followed by weak post-peak fluctuations, often referred to as “ringing” Along the rostro-caudal axis, the peak amplitude gradually decreased, whereas the ringing became more pronounced. Ultimately, the voxel located in the most caudal SC exhibited a nHRF with a significant INR. This voxel-wise analysis revealed a systematic rostro–caudal gradient in HRF temporal dynamics within the SC, characterized by a progressive reduction in peak amplitude and increasingly pronounced post-peak oscillations toward the caudal region.

**Figure 4:**
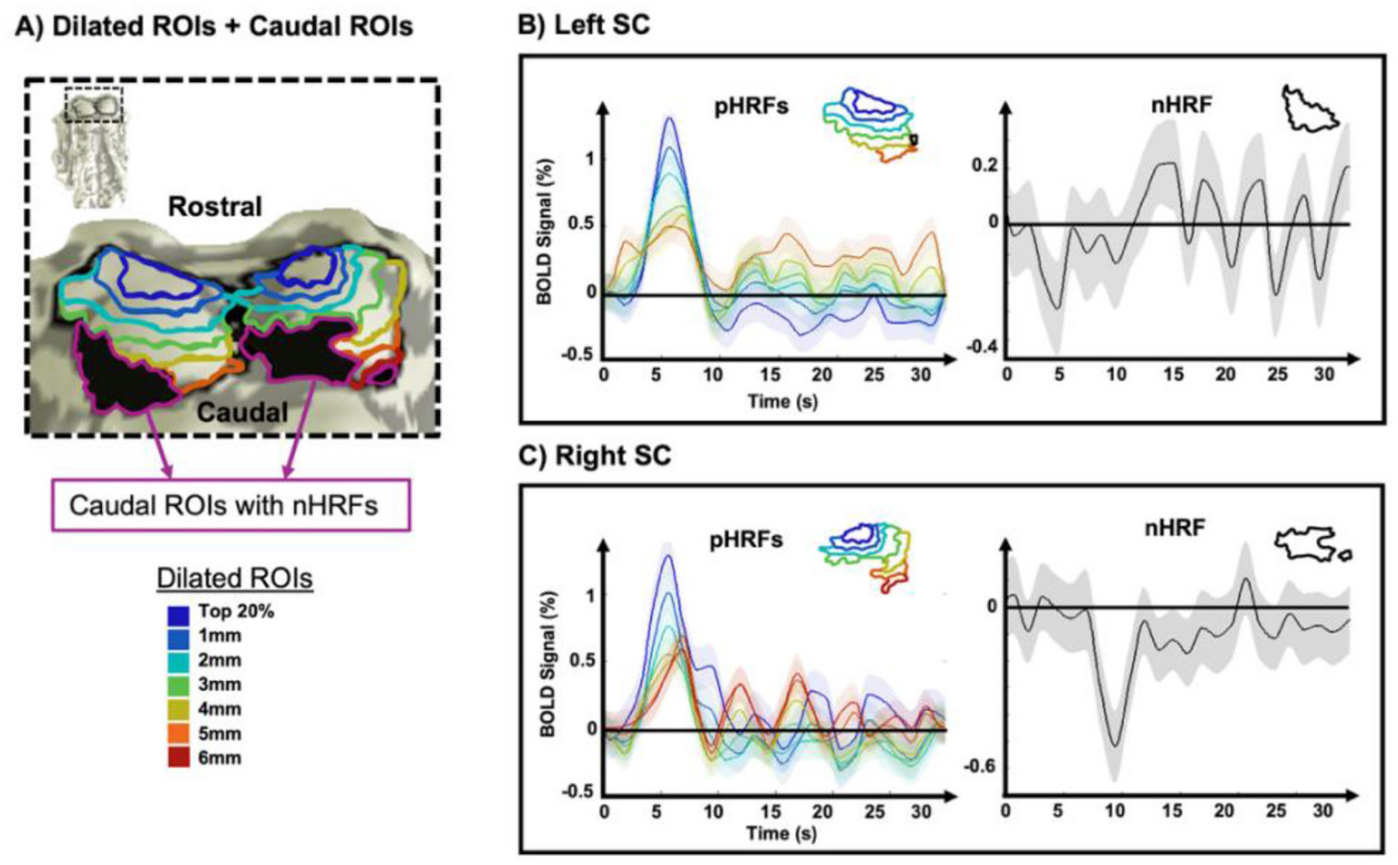
**(A)** 1mm-thick band-shaped ROIs expanded along the rostro-caudal axis in one example subject and corresponding HRFs in the **(B)** left and **(C)** right SC. Peak amplitudes of the pHRF gradually decreased from rostral to caudal SC. Note that strongest HRF was found in most rostral SC while the nHRF was found in caudal SC. The shaded area represents the SEM.

*Dilation-based analysis**:* To further analyze the spatial variation of HRF temporal dynamics in the SC, we created 1-mm-thick, band-shaped ROIs. A different number of dilated ROIs was defined for each SC, reflecting individual variations in its shape and size. For example, in one representative subject, the left SC had five band-shaped ROIs, while the right SC had six (Figure 4A). In this subject, the pHRFs progressively changed caudally, beginning with the most rostral SC ROI that contained strong pHRFs (Figure 4A, dark blue regions). From the most rostral to the most caudal dilated ROIs, the peak amplitude steadily decreased, whereas the ringing became more pronounced (Figures 4B and 4C, left)–consistent with the voxel-wise results. Notably, distinct nHRF dynamics were observed between the left and right caudal SC in this subject: the nHRF in the left SC showed an INR followed by a delayed peak (Figure 4B, right), whereas a substantially delayed INR (~10 s) was observed in the right SC (Figure 4C, right).

**Figure 5:**
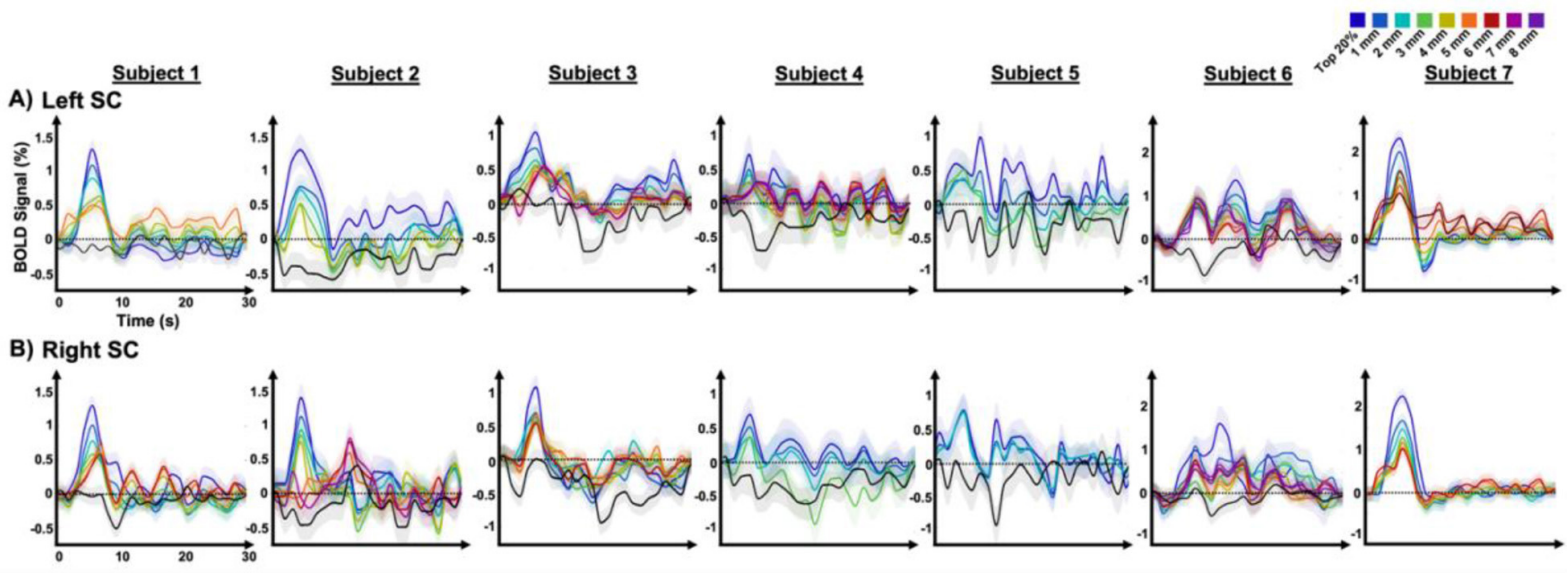
Dilation-based variations in the temporal dynamics of HRFs across subjects for both **(A)** the left, and **(B)** the right SC. The shaded area represents the SEM.

Despite inter-subject variability, these characteristics were consistently observed in all subjects (Figure 5). Although the number of dilated ROIs defined per SC varied both within and across subjects (2–7 range), the dilation analysis showed a consistent attenuation of peak amplitude from rostral to caudal ROIs across both the left and right SCs for all subjects. This variability reflects both anatomical differences in the rostro-caudal extent of the SC and inter-subject differences in the spatial distribution of pHRFs. Because dilation was initiated from the most rostral cluster of strong pHRFs and constrained within the anatomical boundaries of each SC, subjects with a smaller SC or a more restricted pHRF region yielded fewer dilated ROIs. Post-peak ringing was generally increased toward caudal ROIs, though in some cases it was minimal (subject 7 in Figure 5B). Additionally, temporal dynamics of pHRFs in the SC exhibited more deviation from those typically observed in the cortex; in some subjects, strong post-peak ringing with minimal damping was presented (subjects 5 and 6, Figure 5).

Moreover, in all subjects except one (subject 7), the nHRF exhibited a significant INR in the SC (Figure 5, black line); subject 7 showed only pHRFs, with no significant nHRFs detected in either the left or right SC. Notably, this subject demonstrated the strongest pHRF amplitudes among all subjects, reaching 2.3% in the left SC and 2.2% in the right SC. The nHRFs showed pronounced intersubject variability (Figure 5, black line). For example, significant negative responses meeting the predefined classification criterion (CNR >1 for the INR) were observed in the left SC of subjects 2, 4, and 6, and in the right SC of subjects 1, 2, 3, and 5, whereas other subjects showed only marginal negative responses in the caudal SC. Even among significant nHRFs, substantial variability in temporal dynamics was evident.

**Figure 6:**
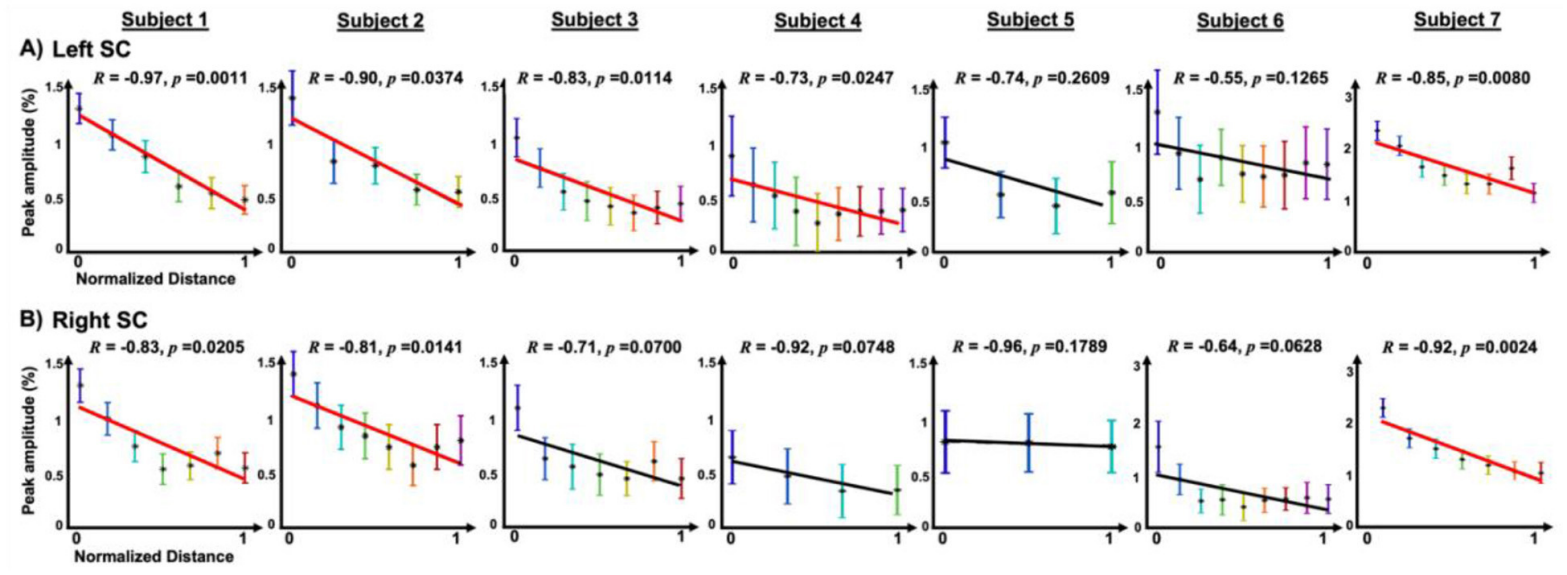
The distributions of peak amplitudes of mean pHRFs across 1mm-dilated ROIs within the rostral pHRF territory (nHRF regions not included) from the most rostral ROI containing the strong pHRFs (normalized distance =0) within both the **(A)** left, and **(B)** right SC for all subjects. The solid red lines show significant (*p* < 0.05) linear regression. R is Pearson correlation coefficient.

Linear regression analysis revealed statistically significant (*p* < 0.05) rostro-caudal trends of decreasing peak amplitudes in 8 of 14 cases (Figure 6). Even in subjects without a significant overall trend, significant differences in peak amplitudes were observed between the most rostral and most caudal ROIs, except for one case (subject 5 in Figure 6B), likely because fewer ROIs were defined along the rostro–caudal axis relative to other subjects.

## Discussion

The SC serves as a critical hub for integrating sensory and motor information to guide visually driven behaviors. Its layered architecture supports distinct functions, while its topographic organization maps visual space, enabling precise control of eye movements across different polar angles and eccentricities (Zubricky and Das, 2025). Notably, previous electrophysiological studies have demonstrated a rostro–caudal representation of eccentricity in the SC, with the rostral SC involved in fixation and small corrective eye movements, and the caudal SC encoding large gaze shifts toward peripheral targets (Gandhi and Katnani, 2011; Hafed et al., 2009; Bergeron and Guitton, 2000; Sugiuchi et al., 2005; Zubricky and Das, 2025). However, such functional specialization in the human SC remains unexplored.

In this study, we systematically characterized the spatiotemporal dynamics of BOLD HRFs within the human SC using ultra–high-field 9.4T MRI combined with a visuomotor task designed to reliably activate collicular responses. We were able to map consistent rostro-caudal evolution of HRFs within the SC. Robust and stereotypical pHRFs were localized to the rostral SC, while their peak amplitudes progressively weakened toward the caudal portion of the SC, eventually giving rise to nHRFs characterized by significant INRs in the most caudal SC. In addition, post-peak ringing was more pronounced toward the caudal SC, further highlighting systematic differences in the HRF temporal dynamics along the rostro-caudal axis. All in all, these findings demonstrate that the human SC's intrinsic topographic organization is reflected not only in the spatial patterns but also in the temporal dynamics of the BOLD HRF, which can be effectively captured using ultra-high field MRI.

The SC exhibits a distinct laminar structure (Benarroch, 2023). Anatomically and functionally, it can be divided into superficial and deep layers (Benarroch, 2023; Liu et al., 2022). The superficial layers receive various afferent inputs originating from the retina and other visual areas, including striate and extrastriate cortices (Stitt et al., 2013; May, 2006; Jiang et al., 2023; Olavarria and Van Sluyters, 1982), forming a topographically organized map of visual field. This map is organized along the medial-lateral axis, representing the upper to lower visual fields, and along the anterior-posterior axis, representing central to peripheral eccentricities. In contrast, the deeper layers receive convergent projections from cortical and subcortical regions associated with multisensory integration, including the frontal and supplementary eye fields, and the primary and supplementary motor cortices (Doykos et al., 2020; Huerta and Kaas, 1990; Liu et al., 2022; Matsumoto et al., 2018). Accordingly, neurons in the deeper layers contribute to oculomotor control, head movements, and stimulus selection (Wall et al., 2009; Zubricky and Das, 2025). Previously, electrophysiological studies in animal models established the laminar functional organization of the SC, underscoring how the SC integrates retinal inputs with multisensory and motor signals to mediate visuomotor and orienting behavior (Durmer and Rosenquist, 2001; Altman and Malis, 1962; White et al., 2019; Bayguinov et al., 2015). This laminar segregation has direct implications for how we approached our analysis. Because our experimental paradigm focused on central visual stimulation, fixation-related oculomotor control, and visual saliency detection, signals from the superficial layers were robustly engaged, whereas deeper-layer activity–normally recruited during large saccades or complex sensorimotor behaviors–was less effectively driven, resulting in weaker or compromised responses. We, therefore, constrained our analysis to more superficial layers, averaging across a depth range of 0.5-2 mm to maximize sensitivity to visually driven responses. This approach also minimized potential contamination from adjacent structures, such as the periaqueductal gray, thereby ensuring that the measured HRFs reflected collicular rather than extraneous midbrain activity (Huneau et al., 2015).

The SC has a critical role in visually guided behaviors, particularly in the initiation and control of saccadic activities (Krauzlis, 2003; Marino et al., 2015; Heeman et al., 2025; Conroy et al., 2024; Soetedjo et al., 2002). The rostro–caudal gradient in BOLD HRF dynamics observed in the SC can be directly related to behavioral demands of our experimental paradigm. In our experimental tasks, subjects were required to perform oculomotor behaviors emphasizing the foveal representation, including fixation, saccades confined to central eccentricities, and pursuit-like tracking, which preferentially engage neurons in the rostral SC (Krauzlis, 2003). Accordingly, strong pHRFs were expected in the rostral SC under these task conditions. By contrast, the caudal SC primarily represents peripheral visual space and large saccades (Gandhi and Katnani, 2011) and was less engaged by our paradigm, resulting in attenuated neural responses. Previous animal studies further indicate that the rostral SC can provide inhibitory inputs to suppress or modulate saccade-related activity originating in the caudal SC, thereby restricting gaze shifts to task-relevant locations (Waitzman, 2005; Sugiuchi et al., 2005; Munoz and Istvan, 1998; Sparks, 1999). Additionally, GABAergic interneurons-either within the SC or projecting to the SC-can selectively refine caudal SC responses (Benarroch, 2023). These inhibitory mechanisms provide a functional framework for interpreting the observed attenuated amplitudes or even nHRFs in the caudal SC. Importantly, although weaker in amplitude, the caudal SC responses were consistent across subjects, suggesting that ultra-high-field fMRI can reliably capture the rostro-caudal gradient of the SC and its functional topography. These findings provide insights into how selectively designed tasks recruit distinct SC subregions in humans, reflecting both spatial (fovea vs. periphery) and behavioral constraints.

Although we classified nHRFs as a distinct group in our analysis, our results indicated that they emerged as part of a gradual evolution rather than as a separate and independent response type. In some subjects, pHRFs progressively transitioned into a transient phase characterized by a more pronounced INR, and ultimately into nHRFs with a significant INR along the rostro–caudal axis (e.g., subject 2, 3 and 4). We have previously studied and reported similar dynamics of the nHRF in the primary visual cortex, where the nHRF gradually evolved as the distance from stimulus representation increased (de la Rosa et al., 2021). Here, in the SC, we also demonstrated that the nHRF is not merely inverted versions of pHRFs (de la Rosa et al., 2021), rather dynamic manifestations of spatially driven HRF transformations.

The vascular and cellular organization of the SC differs from that of the cortex, likely affecting the dynamics of the BOLD HRF. Unlike the stereotypical cortical vascular pattern, in which penetrating arterioles descend into layered circuits, the SC exhibits a more heterogeneous vascular architecture (Duvernoy, 1978; Lee et al., 2022). The SC is primarily supplied by branches of the posterior cerebral artery and, in caudal regions, may also receive input from the superior cerebellar artery (Lee et al., 2022; Niadich et al., 2009). Despite minor variations in arterial supply along the rostro–caudal axis, there is no strong evidence for a systematic gradient in capillary density or venous drainage along this axis. Rather, available anatomical evidence indicates that microvascular organization varies primarily according to laminar structure (Niadich et al., 2009). Nonetheless, previous studies have demonstrated diverse expression patterns of distinct neuronal phenotypes within this complex structure (Xie et al., 2021; Zhang et al., 2019; Shang et al., 2019). Taken together, these findings suggest that the observed systematic rostro–caudal differences in HRF dynamics cannot be fully explained by major differences in vascular anatomy alone and may reflect a combination of vascular and functional factors, including differences in neural response properties or neurovascular coupling. In our previous work, we demonstrated that the subcortical BOLD HRF exhibits faster dynamics than those observed in visual cortex (Kim et al., 2022). Consistently, the present results also revealed similarly fast pHRF dynamics in several subjects (e.g., subjects 1, 2, and 4), further supporting the idea that unique subcortical HRF dynamics arise from the distinct neurovascular and cellular organization of the SC. Notably, some subjects showed patterns that differed from these previous observations (Kim et al., 2022; Wall et al., 2009). This inconsistency may stem from methodological differences: global averaging reflects broad, overall characteristics of the SC, whereas localized analysis can uncover underlying regional heterogeneity in neurovascular and cellular organization.

Our results showed that HRFs can differ in timing across subjects (Figure 5). In addition to inter-subject neurovascular and neurophysiological variability, subtle differences in eye movements or shifts in attention–even during passive viewing–may have influenced SC activation dynamics. Residual effects of head motion or physiological noise could also contribute to HRF timing differences, even after preprocessing. Furthermore, variations in SNR due to slice position, coil sensitivity, or partial volume effects can introduce additional timing variability across subjects (Figure 5) (Krauzlis et al., 2013; Krüger and Glover, 2001; Waitzman, 2005).

Beyond inter-subject variability, we observed subtle temporal differences in HRFs between the left and right SC within individual subjects (Figure 5). These asymmetries may reflect minor variations in vascular anatomy, differences in neural input strength or timing, or localized neurovascular coupling. The SC can also exhibit task-dependent lateralized processing; for example, attention or saccade planning may be dominant in one SC, leading to differences in HRF time-to-peak. Because the SC is a very small structure, even slight differences in ROI placement can sample distinct microvascular or neuronal populations, thereby affecting HRF timing–a well-recognized concern when imaging small brainstem nuclei with fMRI. These intra-subject differences underscore the complexity of subcortical HRF dynamics and highlight the importance of analyzing each colliculus separately rather than averaging across two colliculi (Duvernoy, 1995; Isa and Hall, 2009; Katyal et al., 2012).

We observed systematic changes in post-peak HRF dynamics along the rostro–caudal axis of the SC. Rostral regions showed strong positive responses with mostly minimal post-peak ringing, whereas more caudal regions exhibited progressively larger post-peak ringing that, in some cases, evolved into nHRFs with sustained fluctuations. Although inter-subject variability was evident, the spatial organization of these effects and their reproducibility across subjects argue against a purely noise-driven artifact. Instead, they likely reflect temporally structured neural dynamics and/or neurovascular responses within the SC under the present experimental conditions. The relatively large amplitude of post-peak ringing in the caudal SC may reflect circuit-level interactions within the topographic map. The SC is organized as a competitive sensorimotor structure, with local networks of inhibitory interneurons shaping reciprocal activity between rostral and caudal populations, which help suppressing unwanted saccades while stabilizing fixation (Munoz and Istvan, 1998). Electrophysiological studies have shown that activity in rostral populations can suppress caudal neurons via lateral inhibitory interactions, thereby limiting large gaze shifts when they are not behaviorally required–consistent with traditional inhibitory models of the SC (Waitzman, 2005; Munoz and Istvan, 1998; Sparks, 1999). Such rostro-caudal inhibitory dynamics could shape the temporal profile of population responses, possibly producing delayed suppression or post-inhibitory rebound-like dynamics. At the BOLD level, these neural dynamics—possibly interacting with vascular responses—may manifest as amplified post-peak ringing following diminished peak amplitudes, or even as negative responses in caudal regions.

Thus, the observed nHRFs are most consistent with task-dependent neural suppression than with a measurement-related artifact. Negative BOLD responses have widely associated with reduced neural firing, active inhibition, or redistribution of blood flow toward more strongly activated regions (Shmuel et al., 2006; Devor et al., 2007). In the context of SC, the observed rostro-caudal gradient suggests functional rebalancing across the topographic map, in which strong rostral excitation is accompanied by suppression of caudal populations. Accordingly, the evolution from pHRFs rostrally to attenuated or negative responses caudally is consistent with known competitive and inhibitory circuitry within the SC (Sugiuchi et al., 2005; Benarroch, 2023; Munoz and Istvan, 1998; Waitzman, 2005).

There are several limitations associated with this study. First, our analysis was constrained to superficial layers. This constraint, however, does not weaken overall interpretation of the observed rostro-caudal gradients. In this study, we primarily focused on the superficial SC to maximize sensitivity to visually driven responses, while also avoiding potential contamination from adjacent structures, particularly the periaqueductal gray. Nonetheless, future work with layer-specific fMRI analysis with improved spatiotemporal resolution may provide complementary insight into depth-dependent dynamics. Second, although we classified HRFs into positive and negative groups, our own results indicated that nHRFs are not an independent category but rather emerge gradually from pHRFs along the rostro–caudal axis. This continuous evolution complicates simple binary classification, but at the same time, it highlights the dynamic nature of HRF transformations within the SC. More comprehensive characterization of nHRFs may provide useful insights for systematic analysis without any oversimplification in the future. Third, although the sample size was modest, our experimental design with the repeated-measure design, a large number of HRFs per subject, provided sufficient power to detect robust effects, showing consistent rostro–caudal gradients observed across both SC. The reliability of trends across individuals indicated that our findings are not simply noise-driven but reflect stable features of SC organization. Fourth, our experimental paradigm—in which colored-dot patches were randomly presented at one of three visual-field locations—may not reflect intrinsic functional organization alone but also include contributions from surround suppression. Under surround suppression, one would expect stronger pHRFs rostrally with nHRFs emerging more caudally, a pattern reported in the visual cortex (including our prior work). However, in early visual cortex we did not observe either a consistent gradient from foveal to peripheral representations or reliable peripheral nHRFs (data not shown). In the future, additional experiments using static, centrally presented stimuli with tightly constrained eccentricity and no eye movements could help disentangle contributions from functional organization vs. surround suppression. Finally, ultra-high-field fMRI of small subcortical structures such as the SC has inherent technical challenges, including physiological noise, vascular heterogeneity, and g-factor-related signal-to-noise ratio (SNR) penalties associated with parallel imaging. Consistent with these constraints, temporal SNR within the SC was lower than typically observed in cortical regions (Supplementary Table S1). Nevertheless, visual inspection of the EPI images confirmed preserved signal intensity in the midbrain (Supplementary Figure S2). Together with the observed rostro–caudal gradients and reproducible HRF patterns across scans and subjects, these results indicate that the reported effects are not noise–driven but instead reflect stable functional characteristics of the SC.

In conclusion, this study demonstrates that ultra–high–field fMRI can non-invasively capture detailed functional gradients within the human SC, revealing a systematic rostro–caudal evolution of HRFs from robust pHRFs in the rostral SC to attenuated and even nHRF in the caudal SC. These findings are consistent with organizational principles previously established in animal electrophysiology yet extend them into the human brain by directly linking topographic specialization to hemodynamic dynamics in a subcortical structure, providing insights into SC's neuronal processing in visually-guided behaviors In addition, our work established feasibility of using ultra–high–field MRI to study the neurovascular responses of small and deeply located nuclei, thereby opening various opportunities to non-invasively explore human subcortical function.

## Data Availability

The raw data supporting the conclusions of this article will be made available by the authors, without undue reservation.

## References

[B1] Altman J. Malis L. I. (1962). An electrophysiological study of the superior colliculus and visual cortex. Exp. Neurol. 5, 233–249. doi: 10.1016/0014-4886(62)90036-5 13860745

[B2] Bajaj C. L. Xu G. Zhang Q. (2008a). Higher-order level-set method and its application in biomolecular surfaces construction. J. Comput. Sci. Technol. 23, 1026–1036. doi: 10.0000/j.1000-9000.200823610261036 20495682 PMC2873780

[B3] Bajaj C. L. Xu G.-L. Zhang Q. (2008b). Bio-molecule surfaces construction via a higher-order level-set method. J. Comput. Sci. Technol. 23, 1026–1036. doi: 10.1007/s11390-008-9184-1 20495682 PMC2873780

[B4] Barry R. L. Coaster M. Rogers B. P. Newton A. T. Moore J. Anderson A. W. . (2013). On the origins of signal variance in FMRI of the human midbrain at high field. PLoS ONE 8:e62708. doi: 10.1371/journal.pone.0062708 23658643 PMC3637217

[B5] Bayguinov P. O. Ghitani N. Jackson M. B. Basso M. A. (2015). A hard-wired priority map in the superior colliculus shaped by asymmetric inhibitory circuitry. J. Neurophysiol. 114, 662–676. doi: 10.1152/jn.00144.2015 25995346 PMC4512250

[B6] Benarroch E. (2023). What are the functions of the superior colliculus and its involvement in neurologic disorders? Neurology 100, 784–790. doi: 10.1212/WNL.0000000000207254 37068960 PMC10115501

[B7] Benavidez N. L. Bienkowski M. S. Zhu M. Garcia L. H. Fayzullina M. Gao L. . (2021). Organization of the inputs and outputs of the mouse superior colliculus. Nat. Commun. 12:4004. doi: 10.1038/s41467-021-24241-2 34183678 PMC8239028

[B8] Bergeron A. Guitton D. (2000). Fixation neurons in the superior colliculus encode distance between current and desired gaze positions. Nat. Neurosci. 3, 932–939. doi: 10.1038/78847 10966625

[B9] Boynton G. M. Engel S. A. Glover G. H. Heeger D. J. (1996). Linear systems analysis of functional magnetic resonance imaging in human V1. J. Neurosci. 16, 4207–4221. doi: 10.1523/JNEUROSCI.16-13-04207.1996 8753882 PMC6579007

[B10] Burnett L. R. Stein B. E. Chaponis D. Wallace M. T. (2004). Superior colliculus lesions preferentially disrupt multisensory orientation. Neuroscience 124, 535–547. doi: 10.1016/j.neuroscience.2003.12.026 14980725

[B11] Conroy C. Nanjappa R. McPeek R. M. (2024). Inhibitory tagging both speeds and strengthens saccade target selection in the superior colliculus during visual search. J. Neurophysiol. 131, 548–555. doi: 10.1152/jn.00355.2023 38292000 PMC11305629

[B12] Dale A. M. Fischl B. Sereno M. I. (1999). Cortical surface-based analysis. I. Segmentation and surface reconstruction. NeuroImage 9, 179–194. doi: 10.1006/nimg.1998.0395 9931268

[B13] de la Rosa N. Ress D. Taylor A. J. Kim J. H. (2021). Retinotopic variations of the negative blood-oxygen-level dependent hemodynamic response function in human primary visual cortex. J. Neurophysiol. 125, 1045–1057. doi: 10.1152/jn.00676.2020 33625949 PMC8282228

[B14] Devor A. Tian P. Nishimura N. Teng I. C. Hillman E. M. C. Narayanan S. N. . (2007). Suppressed neuronal activity and concurrent arteriolar vasoconstriction may explain negative blood oxygenation level-dependent signal. J. Neurosci. 27, 4452–4459. doi: 10.1523/jneurosci.0134-07.2007 17442830 PMC2680207

[B15] Doykos T. K. Gilmer J. I. Person A. L. Felsen G. (2020). Monosynaptic inputs to specific cell types of the intermediate and deep layers of the superior colliculus. J. Comp. Neurol. 528, 2254–2268. doi: 10.1002/cne.24888 32080842 PMC8032550

[B16] Durmer J. S. Rosenquist A. C. (2001). Ibotenic acid lesions in the pedunculopontine region result in recovery of visual orienting in the hemianopic cat. Neuroscience 106, 765–781. doi: 10.1016/s0306-4522(01)00321-9 11682162

[B17] Duvernoy H. M. (1978). Human Brainstem Vessels. Berlin; Heidelberg: Springer. doi: 10.1007/978-3-662-02299-3

[B18] Duvernoy H. M. (1995). “Anatomy of the brain stem and cerebellum surfaces,” in The Human Brain Stem and Cerebellum (Vienna: Springer). doi: 10.1007/978-3-7091-3078-0_3

[B19] Düzel E. Guitart-Masip M. Maass A. Hämmerer D. Betts M. J. Speck O. . (2015). “Midbrain fMRI: applications, limitations and challenges,” *in fMRI: From Nuclear Spins to Brain Functions*, eds. K. Uludag, K. Ugurbil, and L. Berliner (Springer), 581–609. doi: 10.1007/978-1-4899-7591-1_20

[B20] Efron B. (1981). Nonparametric estimates of standard error: the Jackknife, the Bootstrap and other methods. Biometrika 68, 589–599. doi: 10.2307/2335441

[B21] Efron B. (1987). Better bootstrap confidence intervals. J. Am. Stat. Assoc. 82, 171–185. doi: 10.1080/01621459.1987.10478410

[B22] Efron B. Tibshirani R. J. (1994). An Introduction to the Bootstrap. Chapman and Hall/CRC. doi: 10.1201/9780429246593

[B23] Fesharaki N. J. Taylor A. Mosby K. Li R. Kim J. H. Ress D. (2024). Global impact of aging on the hemodynamic response function in the gray matter of human cerebral cortex. Hum. Brain Mapp. 45:e70100. doi: 10.1002/hbm.70100 39692126 PMC11653092

[B24] Fesharaki N. J. Taylor A. Ress D. (2026). Age-related variations of the hemodynamic response function spatially resolved across human cerebral cortex. Front. Aging Neurosci. 18:1774543. doi: 10.3389/fnagi.2026.1774543 41822298 PMC12975896

[B25] Field C. B. Johnston K. Gati J. S. Menon R. S. Everling S. (2008). Connectivity of the primate superior colliculus mapped by concurrent microstimulation and event-related fMRI. PLoS ONE 3:e3928. doi: 10.1371/journal.pone.0003928 19079541 PMC2592545

[B26] Gandhi N. J. Katnani H. A. (2011). Motor functions of the superior colliculus. Annu. Rev. Neurosci. 34, 205–231. doi: 10.1146/annurev-neuro-061010-113728 21456962 PMC3641825

[B27] Gil R. Valente M. Shemesh N. (2024). Rat superior colliculus encodes the transition between static and dynamic vision modes. Nat. Commun. 15:849. doi: 10.1038/s41467-024-44934-8 38346973 PMC10861507

[B28] Hafed Z. M. Goffart L. Krauzlis R. J. (2009). A neural mechanism for microsaccade generation in the primate superior colliculus. Science (New York, N.Y.) 323, 940–943. doi: 10.1126/science.1166112 19213919 PMC2655118

[B29] Heeman J. White B. J. Van der Stigchel S. Theeuwes J. Itti L. Munoz D. P. (2025). Saliency response in superior colliculus at the future saccade goal predicts fixation duration during free viewing of dynamic scenes. J. Neurosci. 45:e0428242024. doi: 10.1523/JNEUROSCI.0428-24.2024 39572235 PMC11735649

[B30] Himmelbach M. Erb M. Karnath H.-O. (2007). Activation of superior colliculi in humans during visual exploration. BMC Neurosci. 8:66. doi: 10.1186/1471-2202-8-66 17697355 PMC1976416

[B31] Huerta M. F. Kaas J. H. (1990). Supplementary eye field as defined by intracortical microstimulation: Connections in macaques. J. Comp. Neurol. 293, 299–330. doi: 10.1002/cne.902930211 19189718

[B32] Huneau C. Benali H. Chabriat H. (2015). Investigating human neurovascular coupling using functional neuroimaging: a critical review of dynamic models. Front. Neurosci. 9:467. doi: 10.3389/fnins.2015.00467 26733782 PMC4683196

[B33] Iglesias J. E. Van Leemput K. Bhatt P. Casillas C. Dutt S. Schuff N. . (2015). Bayesian segmentation of brainstem structures in MRI. NeuroImage 113, 184–195. doi: 10.1016/j.neuroimage.2015.02.065 25776214 PMC4434226

[B34] Ikeda T. Hikosaka O. (2007). Positive and negative modulation of motor response in primate superior colliculus by reward expectation. J. Neurophysiol. 98, 3163–3170. doi: 10.1152/jn.00975.2007 17928551

[B35] Isa T. Hall W. C. (2009). Exploring the superior colliculus *in vitro*. J. Neurophysiol. 102, 2581–2593. doi: 10.1152/jn.00498.2009 19710376 PMC2777828

[B36] Jiang S. Honnuraiah S. Stuart G. J. (2023). Characterization of primary visual cortex input to specific cell types in the superior colliculus. Front. Neuroanat. 17:1282941. doi: 10.3389/fnana.2023.1282941 38020214 PMC10667433

[B37] Jun E. J. Bautista A. R. Nunez M. D. Allen D. C. Tak J. H. Alvarez E. . (2021). Causal role for the primate superior colliculus in the computation of evidence for perceptual decisions. Nat. Neurosci. 24, 1121–1131. doi: 10.1038/s41593-021-00878-6 34183869 PMC8338902

[B38] Katyal S. Greene C. A. Ress D. (2012). High-resolution functional magnetic resonance imaging methods for human midbrain. JoVE 63:e3746. doi: 10.3791/3746-v 22617680 PMC3468195

[B39] Katyal S. Ress D. (2014). Endogenous attention signals evoked by threshold contrast detection in human superior colliculus. J. Neurosci. 34, 892–900. doi: 10.1523/JNEUROSCI.3026-13.2014 24431447 PMC6608350

[B40] Katyal S. Zughni S. Greene C. Ress D. (2010). Topography of covert visual attention in human superior colliculus. J. Neurophysiol. 104, 3074–3083. doi: 10.1152/jn.00283.2010 20861435

[B41] Khan R. Zhang Q. Darayan S. Dhandapani S. Katyal S. Greene C. . (2011). Surface-based analysis methods for high-resolution functional magnetic resonance imaging. Graph. Models 73, 313–322. doi: 10.1016/j.gmod.2010.11.002 22125419 PMC3223917

[B42] Kim B. Basso M. A. (2008). Saccade target selection in the superior colliculus: a signal detection theory approach. J. Neurosci. 28, 2991–3007. doi: 10.1523/JNEUROSCI.5424-07.2008 18354003 PMC6670709

[B43] Kim J. H. Ress D. (2017). Reliability of the depth-dependent high-resolution BOLD hemodynamic response in human visual cortex and vicinity. Magn. Reson. Imaging 39, 53–63. doi: 10.1016/j.mri.2017.01.019 28137626 PMC5410406

[B44] Kim J. H. Taylor A. J. Himmelbach M. Hagberg G. E. Scheffler K. Ress D. (2022). Characterization of the blood oxygen level dependent hemodynamic response function in human subcortical regions with high spatiotemporal resolution. Front. Neurosci. 16:1009295. doi: 10.3389/fnins.2022.1009295 36303946 PMC9592726

[B45] Krauzlis R. J. (2003). Neuronal activity in the rostral superior colliculus related to the initiation of pursuit and saccadic eye movements. J. Neurosci. 23, 4333–4344. doi: 10.1523/JNEUROSCI.23-10-04333.2003 12764122 PMC6741111

[B46] Krauzlis R. J. Lovejoy L. P. Zénon A. (2013). Superior colliculus and visual spatial attention. Annu. Rev. Neurosci. 36, 165–182. doi: 10.1146/annurev-neuro-062012-170249 23682659 PMC3820016

[B47] Krebs R. M. Woldorff M. G. Tempelmann C. Bodammer N. Noesselt T. Boehler C. N. . (2010). High-field fMRI reveals brain activation patterns underlying saccade execution in the human superior colliculus. PLoS ONE 5:e8691. doi: 10.1371/journal.pone.0008691 20084170 PMC2805712

[B48] Krüger G. Glover G. H. (2001). Physiological noise in oxygenation-sensitive magnetic resonance imaging. Magn. Reson. Med. 46, 631–637. doi: 10.1002/mrm.1240 11590638

[B49] Lau C. Zhang J. W. Xing K. K. Zhou I. Y. Cheung M. M. Chan K. C. . (2011). BOLD responses in the superior colliculus and lateral geniculate nucleus of the rat viewing an apparent motion stimulus. NeuroImage 58, 878–884. doi: 10.1016/j.neuroimage.2011.06.055 21741483

[B50] Lee J. Y. Mack A. F. Shiozawa T. Longo R. Tromba G. Scheffler K. . (2022). Microvascular imaging of the unstained human superior colliculus using synchrotron-radiation phase-contrast microtomography. Sci. Rep. 12:9238. doi: 10.1038/s41598-022-13282-2 35655082 PMC9163179

[B51] Lewis L. D. Setsompop K. Rosen B. R. Polimeni J. R. (2018). Stimulus-dependent hemodynamic response timing across the human subcortical-cortical visual pathway identified through high spatiotemporal resolution 7T fMRI. NeuroImage 181, 279–291. doi: 10.1016/j.neuroimage.2018.06.056 29935223 PMC6245599

[B52] Limbrick-Oldfield E. H. Brooks J. C. W. Wise R. J. S. Padormo F. Hajnal J. V. Beckmann C. F. . (2012). Identification and characterisation of midbrain nuclei using optimised functional magnetic resonance imaging. NeuroImage 59, 1230–1238. doi: 10.1016/j.neuroimage.2011.08.016 21867762 PMC3236997

[B53] Liu X. Huang H. Snutch T. P. Cao P. Wang L. Wang F. (2022). The superior colliculus: cell types, connectivity, and behavior. Neurosci. Bull. 38, 1519–1540. doi: 10.1007/s12264-022-00858-1 35484472 PMC9723059

[B54] Logothetis N. K. Pauls J. Augath M. Trinath T. Oeltermann A. (2001). Neurophysiological investigation of the basis of the fMRI signal. Nature 412, 150–157. doi: 10.1038/35084005 11449264

[B55] Lovejoy L. P. Krauzlis R. J. (2010). Inactivation of primate superior colliculus impairs covert selection of signals for perceptual judgments. Nat. Neurosci. 13, 261–266. doi: 10.1038/nn.2470 20023651 PMC3412590

[B56] Marino R. A. Levy R. Munoz D. P. (2015). Linking express saccade occurance to stimulus properties and sensorimotor integration in the superior colliculus. J. Neurophysiol. 114, 879–892. doi: 10.1152/jn.00047.2015 26063770 PMC4533109

[B57] Matsumoto M. Inoue K. Takada M. (2018). Causal role of neural signals transmitted from the frontal eye field to the superior colliculus in saccade generation. Front. Neural Circuits 12:69. doi: 10.3389/fncir.2018.00069 30210307 PMC6120992

[B58] May P. J. (2006). The mammalian superior colliculus: laminar structure and connections. Prog. Brain Res. 151, 321–378. doi: 10.1016/S0079-6123(05)51011-2 16221594

[B59] Moro E. Bellot E. Meoni S. Pelissier P. Hera R. Dojat M. . (2020). Visual dysfunction of the superior colliculus in de novo Parkinsonian patients. Ann. Neurol. 87, 533–546. doi: 10.1002/ana.25696 32030799

[B60] Munoz D. P. Istvan P. J. (1998). Lateral inhibitory interactions in the intermediate layers of the monkey superior colliculus. J. Neurophysiol. 79, 1193–1209. doi: 10.1152/jn.1998.79.3.1193 9497401

[B61] Nestares O. Heeger D. J. (2000). Robust multiresolution alignment of MRI brain volumes. Magn. Reson. Med. 43, 705–715. doi: 10.1002/(SICI)1522-2594(200005)43:5<705::AID-MRM13>3.0.CO;2-R 10800036

[B62] Niadich T. P. Duvernoy H. M. Delman B. N. Sorensen A. G. Kollias S. S. Haacke E. M. (2009). Duvernoy's Atlas of the Human Brain Stem and Cerebellum: High-Field MRI, Surface Anatomy, Internal Structure, Vascularization and 3 D Sectional Anatomy. Wien: Springer. Available online at: https://www.abebooks.com/9783211739709/Duvernoys-Atlas-Human-Brain-Stem-321173970X/plp (Accessed April 7, 2026).

[B63] Ogawa S. Tank D. W. Menon R. Ellermann J. M. Kim S. G. Merkle H. . (1992). Intrinsic signal changes accompanying sensory stimulation: functional brain mapping with magnetic resonance imaging. Proc. Natl. Acad. Sci. U S A. 89, 5951–5955. doi: 10.1073/pnas.89.13.5951 1631079 PMC402116

[B64] Olavarria J. Van Sluyters R. C. (1982). The projection from striate and extrastriate cortical areas to the superior colliculus in the rat. Brain Res. 242, 332–336. doi: 10.1016/0006-8993(82)90318-3 6180800

[B65] Savjani R. R. Katyal S. Halfen E. Kim J. H. Ress D. (2018). Polar-angle representation of saccadic eye movements in human superior colliculus. NeuroImage 171, 199–208. doi: 10.1016/j.neuroimage.2017.12.080 29292132 PMC6844626

[B66] Schneider K. A. Kastner S. (2005). Visual responses of the human superior colliculus: a high-resolution functional magnetic resonance imaging study. J. Neurophysiol. 94, 2491–2503. doi: 10.1152/jn.00288.2005 15944234

[B67] Schumann A. Köhler S. de la Cruz F. Güllmar D. Reichenbach J. R. Wagner G. . (2018). The use of physiological signals in brainstem/midbrain fMRI. Front. Neurosci. 12:718. doi: 10.3389/fnins.2018.00718 30386203 PMC6198067

[B68] Shang C. Liu A. Li D. Xie Z. Chen Z. Huang M. . (2019). A subcortical excitatory circuit for sensory-triggered predatory hunting in mice. Nat. Neurosci. 22, 909–920. doi: 10.1038/s41593-019-0405-4 31127260

[B69] Shinkareva S. V. Wang J. Kim J. Facciani M. J. Baucom L. B. Wedell D. H. (2013). Representations of modality-specific affective processing for visual and auditory stimuli derived from functional magnetic resonance imaging data. Hum. Brain Mapp. 35, 3558–3568. doi: 10.1002/hbm.22421 24302696 PMC6869138

[B70] Shmuel A. Augath M. Oeltermann A. Logothetis N. K. (2006). Negative functional MRI response correlates with decreases in neuronal activity in monkey visual area V1. Nat. Neurosci. 9, 569–577. doi: 10.1038/nn1675 16547508

[B71] Soetedjo R. Kaneko C. R. S. Fuchs A. F. (2002). Evidence that the superior colliculus participates in the feedback control of saccadic eye movements. J. Neurophysiol. 87, 679–695. doi: 10.1152/jn.00886.2000 11826037

[B72] Sparks D. L. (1999). Conceptual issues related to the role of the superior colliculus in the control of gaze. Curr. Opin. Neurobiol. 9, 698–707. doi: 10.1016/S0959-4388(99)00039-2 10607648

[B73] Stitt I. Galindo-Leon E. Pieper F. Engler G. Engel A. K. (2013). Laminar profile of visual response properties in ferret superior colliculus. J. Neurophysiol. 110, 1333–1345. doi: 10.1152/jn.00957.2012 23803328

[B74] Sugiuchi Y. Izawa Y. Takahashi M. Na J. Shinoda Y. (2005). Physiological characterization of synaptic inputs to inhibitory burst neurons from the rostral and caudal superior colliculus. J. Neurophysiol. 93, 697–712. doi: 10.1152/jn.00502.2004 15653784

[B75] Taylor A. J. Kim J. H. Ress D. (2018). Characterization of the hemodynamic response function across the majority of human cerebral cortex. NeuroImage 173, 322–331. doi: 10.1016/j.neuroimage.2018.02.061 29501554 PMC5911213

[B76] Teo P. C. Sapiro G. Wandell B. A. (1997). Creating connected representations of cortical gray matter for functional MRI visualization. IEEE Trans. Med. Imaging 16, 852–863. doi: 10.1109/42.650881 9533585

[B77] Truong P. Kim J. H. Savjani R. Sitek K. R. Hagberg G. E. Scheffler K. . (2020). Depth relationships and measures of tissue thickness in dorsal midbrain. Hum. Brain Mapp. 41, 5083–5096. doi: 10.1002/hbm.25185 32870572 PMC7670631

[B78] Waitzman D. M. (2005). So much to see, so little time: How the superior colliculus (SC) suppresses unwanted saccades. Focus on “Physiological characterization of synaptic inputs to inhibitory burst neurons from the rostral and caudal superior colliculus.” J. Neurophysiol. 93, 641–642. doi: 10.1152/jn.00977.2004 15653782

[B79] Wall M. B. Walker R. Smith A. T. (2009). Functional imaging of the human superior colliculus: an optimised approach. NeuroImage 47, 1620–1627. doi: 10.1016/j.neuroimage.2009.05.094 19505584

[B80] Wang Y. C. Bianciardi M. Chanes L. Satpute A. B. (2020). Ultra high field fMRI of human superior colliculi activity during affective visual processing. Sci. Rep. 10:1331. doi: 10.1038/s41598-020-57653-z 31992744 PMC6987103

[B81] White B. J. Kan J. Y. Itti L. Munoz D. P. (2019). Laminar organization of the superior colliculus priority map. J. Vis. 19:133a. doi: 10.1167/19.10.133a

[B82] World Medical Association (2013). World Medical Association Declaration of Helsinki: ethical principles for medical research involving human subjects. JAMA 310, 2191–2194. doi: 10.1001/jama.2013.281053 24141714

[B83] Xie Z. Wang M. Liu Z. Shang C. Zhang C. Sun L. . (2021). Transcriptomic encoding of sensorimotor transformation in the midbrain. eLife 10:e69825. doi: 10.7554/eLife.69825 34318750 PMC8341986

[B84] Xu G. Pan Q. Bajaj C. L. (2006). Discrete surface modelling using partial differential equations. Comput. Aided Geom. Des. 23, 125–145. doi: 10.1016/j.cagd.2005.05.004 19830268 PMC2760856

[B85] Yushkevich P. A. Yang G. Gerig G. (2016). ITK-SNAP: An interactive tool for semi-automatic segmentation of multi-modality biomedical images. Annu. Int. Conf. IEEE Eng. Med. Biol. Soc. 2016, 3342–3345. doi: 10.1109/EMBC.2016.7591443 28269019 PMC5493443

[B86] Zhang Z. Liu W.-Y. Diao Y.-P. Xu W. Zhong Y.-H. Zhang J.-Y. . (2019). Superior colliculus GABAergic neurons are essential for acute dark induction of wakefulness in mice. Curr. Biol. 29, 637–644.e3. doi: 10.1016/j.cub.2018.12.031 30713103

[B87] Zubricky R. D. Das J. M. (2025). “Neuroanatomy, superior colliculus,” in StatPearls. StatPearls Publishing. Available online at: http://www.ncbi.nlm.nih.gov/books/NBK544224/ (Accessed April 7, 2026).

