# Correction: Spatial evolution in temporal dynamics of hemodynamic response function in human superior colliculi with ultra-high-resolution MRI at 9.4T

**DOI:** 10.3389/fnins.2026.1911255

**Published:** 2026-07-13

**Authors:** Nooshin J. Fesharaki, Artemy Vinogradov, David Ress, Jung Hwan Kim

**Affiliations:** 1 Department of Neurosurgery, University of Texas Health Science Center at Houston, Houston, TX, United States; 2 Department of Neuroscience, Baylor College of Medicine, Houston, TX, United States

**Keywords:** blood oxygenation level dependent, BOLD positive HRFs, functional magnetic resonance imaging, hemodynamic response function, negative HRF, neurovascular dynamics, spatial gradient, superior colliculi

The **Acknowledgements** section was missing in the original published manuscript. The **Acknowledgments** should be as follows:

We gratefully acknowledge Prof. Dr. Klaus Scheffler at the Max Planck Institute for Biological Cybernetics in Tübingen, Germany, for his thoughtful advice and support for this project. We also thank Dr. Gisela Hagberg and Dr. Marc Himmelbach for their helpful contributions to data collection and guidance. In addition, we acknowledge Dr. Francesko Molla for his assistance with data acquisition. The data were collected at the Max Planck Institute for Biological Cybernetics in Tübingen, Germany.

The original version of this article has been updated.

