# Supplementary material for: Spatial evolution in temporal dynamics of hemodynamic response function in human superior colliculi with ultra-high-resolution MRI at 9.4T
Source: Front Neurosci. 2026 Apr 16;20:1741923. doi: 10.3389/fnins.2026.1741923 (PMC13128421; doi:10.3389/fnins.2026.1741923)
Supplement: Supplementary file 1 [file Data_Sheet_1.pdf]

Spatial evolution in temporal dynamics of hemodynamic response function in human superior colliculi with ultra-high-resolution MRI at 9.4T

Nooshin J. Fesharaki[1], Artemy Vinogradov[1], David Ress[2], Jung Hwan Kim[1*]

[1]Neurosurgery Dept. University of Texas Health Science Center at Houston, TX, USA

[2]Neuroscience Dept. Baylor College of Medicine, Houston, TX, USA

Supplementary materials

Registration quality and superior colliculus (SC) localization

The close correspondence between functional and anatomical boundaries in the midbrain confirms accurate registration and appropriate anatomical localization of the SC for subsequent analyses (Fig. S1). Axial, sagittal, and coronal views are shown to illustrate alignment across imaging planes.

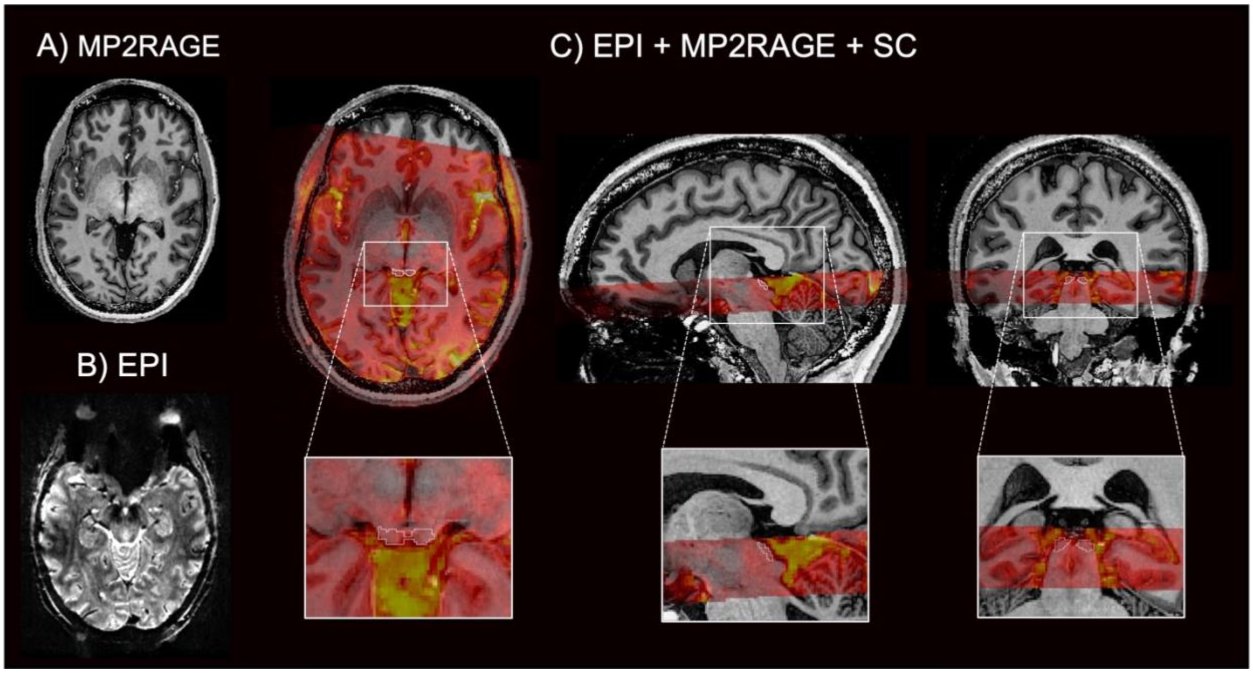

Figure S1. (**A**) High-resolution MP2RAGE anatomical image (axial view). (**B**) Echo planer imaging (EPI) image in functional space (axial view). (**C**) Overlay of the mean EPI image onto the MP2RAGE anatomy, with delineation of the superior colliculus (SC) boundary shown in axial, sagittal, and coronal views.

Visual inspection of Echo-planer Imaging (EPI) images

For each subject, we obtained one representative EPI volume (mean EPI image across time), including axial, sagittal, and coronal views centered on the SC (Fig. S2), demonstrating sufficient signal quality and anatomical delineation at the level of SC for further analysis.

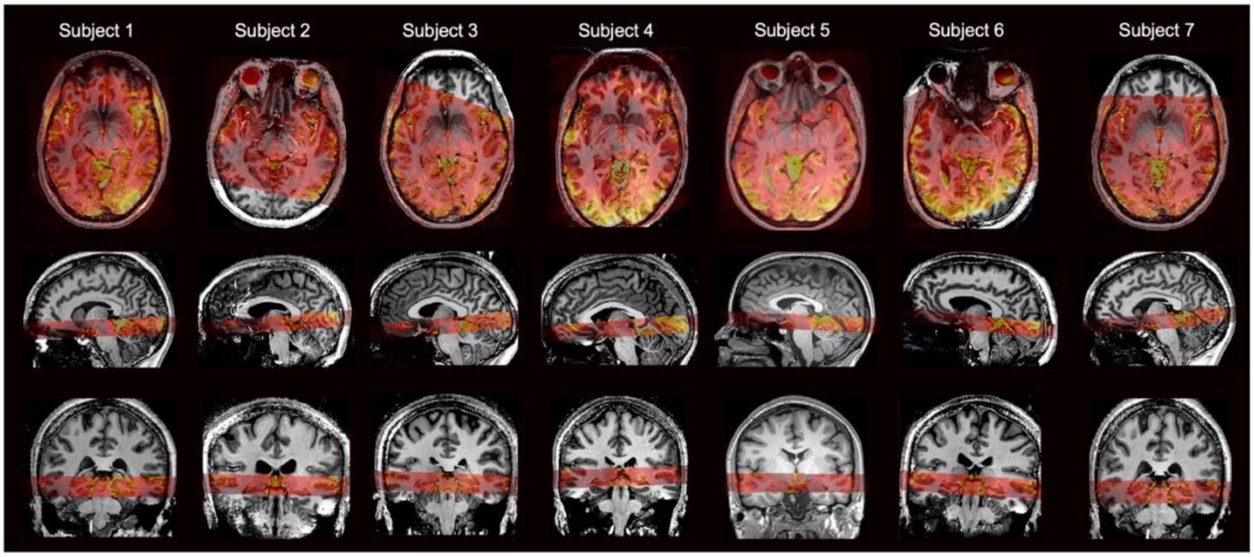

Figure S2. One representative EPI volume per subject in axial, sagittal, and coronal views centered on the subject's superior colliculus.

Temporal signal-to-noise ratio (SNR)

We computed temporal SNR for each voxel within the superior colliculus as the ratio of the mean signal across time to its standard deviation across time. For each subject, SC temporal SNR values were then averaged across scans and subsequently across subjects (Table S1). Individual scan-level temporal SNR values ranged from 4–36.8, and the mean SC temporal SNR across subjects was 10.8 ± 2.8 (range: 4.7–25). These values were lower than those typically reported for cortical regions. However, they are consistent with ultra-high field (9.4T) acquisitions at 1-mm isotropic resolution with GRAPPA acceleration (factor 4), where physiological noise and g-factor–related penalties substantially limit temporal stability, particularly in small midbrain structures such as the SC. Importantly, despite these constraints, SC responses were spatially organized and reproducible across scans and subjects, supporting the reliability of the study's findings.

Table S1. Temporal SNR within the superior colliculus across subjects

	Subjects						
	**1**	**2**	**3**	**4**	**5**	**6**	**7**
**Mean ± Std**	12.5 ± 3.4	12.4 ± 2.6	10 ± 1.9	10.8 ± 2.6	10.1 ± 2.5	10.4 ± 2.7	9.3 ± 1.6
**Range**	4.2–32	4–22.8	4.4–21.6	5.5–22.1	4.4–36.8	4.1–31.3	4.2–19.7
